# Recruitment Strategies for Lung Cancer Screening: An Umbrella Review Protocol

**DOI:** 10.12688/hrbopenres.14160.1

**Published:** 2025-06-25

**Authors:** Bethany Cushing, Benjamin Jacob, XinYi Low, Sam McGlynn, Ildiko Horvath, Martina Vranka, Tytti Sarkeala, Patrick Redmond

**Affiliations:** 1 Royal College of Surgeons in Ireland School of Medicine, Dublin, Leinster, Ireland; 2 Royal College of Surgeons in Ireland Department of General Practice, Dublin, Leinster, Ireland; 3 National Koranyi Institute for Pulmonology, Budapest, Hungary; 4 Division of Radiotherapy, Institute of Oncology Ljubljana, Ljubljana, Slovenia; 5 Finnish Cancer Registry, Helsinki, 00500, Finland

**Keywords:** Umbrella review; Early detection of cancer; Cancer screening; Lung cancer; participation; Primary care

## Abstract

**Background:**

Lung cancer is the leading cause of cancer-related mortality worldwide. Low-dose CT (LDCT) lung cancer screening (LCS) reduces lung cancer-specific mortality by 20%, yet participation remains low, often below 15%, compared with 60–75% for other cancer screening programmes. Barriers such as limited accessibility, stigma, fear of diagnosis, and misconceptions contribute to poor uptake, particularly among high-risk groups, including heavy smokers, ethnic minorities, and individuals from lower socioeconomic backgrounds. Various recruitment strategies—such as personalised invitations, media campaigns, and primary care referrals—have been implemented, but their effectiveness across different populations remains unclear. This umbrella review will synthesise evidence from systematic reviews to identify the most effective recruitment strategies for improving LCS participation.

**Methods:**

This umbrella review will follow Joanna Briggs Institute guidelines and the PRIOR reporting framework. A systematic search of PubMed, Embase, Scopus, Web of Science, Cochrane Library, and systematic review registries will identify systematic reviews published before 31 October 2024. Eligible reviews must evaluate LCS recruitment strategies and report on at least one of the following: population reach, screening up take, adherence, patient experience, or implementation barriers. Quality will be assessed using AMSTAR 2, and overlapping primary studies will be mapped to prevent duplication. A narrative synthesis will categorise recruitment strategies, and a qualitative effectiveness ranking will summarise key findings.

**Implications:**

Findings will inform LCS recruitment strategies in Europe, contributing to the EU4Health-funded EUCanScreen programme. This review will support efforts to improve uptake, reduce disparities, and enhance early detection and survival outcomes of lung cancer.

## Introduction

### Lung cancer and the role of screening

Lung cancer is the leading cause of cancer-related mortality worldwide, accounting for approximately 1.8 million deaths annually ^1^. Despite advances in treatment, five‐year survival rates continue to range between 10–20%, largely owing to late-stage diagnosis ^2, 3^. Low-dose computed tomography (LDCT) lung cancer screening (LCS) has emerged as an effective intervention; controlled trials have demonstrated that LDCT screening can reduce lung cancer-specific mortality by 20% ^2, 3^. As a result, multiple international guidelines now recommend risk-stratified LDCT screening for high-risk individuals, typically long-term smokers ^4^.

The United States Preventive Services Task Force (USPSTF) recommends annual LDCT screening for adults aged 50 to 80 years with a minimum 20 pack-year smoking history, provided they currently smoke or have quit within the past 15 years ^5^. Similarly, the European Commission’s 2022 Council Recommendation prioritises the implementation of LCS pilot programmes across EU member states, recognising the need for targeted recruitment strategies to optimise participation.

### Global policy and implementation challenges

Despite robust evidence supporting LDCT screening, its implementation remains fragmented, with substantial disparities in screening availability and participation across different healthcare systems. The United States has been at the forefront of LCS policy development, with structured screening programmes integrated into national guidelines. However, even within well-established screening systems, participation remains low, often below 5% of eligible individuals ^4^.

In contrast, European countries have been more cautious; only a handful—such as Poland, Croatia, and Italy—have committed to nationwide LCS initiatives. Most European nations rely on opportunistic recruitment, often through primary care or hospital-based settings, contributing to low participation rates. In Asia and other regions, the lack of organised screening programmes, coupled with epidemiological differences—such as a higher proportion of lung cancer cases among never-smokers—further complicates the adoption of standardised screening criteria ^6^.

Beyond technical and policy issues, infrastructural constraints such as limited CT scanner availability, insufficient radiology expertise, and inadequate follow-up systems further impede large-scale implementation. Financial constraints and lack of reimbursement policies further exacerbate disparities, particularly in lower-resource settings ^7^. These challenges have been amplified by strains on healthcare systems following the COVID-19 pandemic, delaying progress in LCS programme development ^7^.

***Disparities in screening participation***

Even within countries where LCS guidelines are in place, participation remains critically low. Whereas breast and colorectal cancer screening programmes often achieve participation rates of 60–75%, lung cancer screening rates are typically below 15% ^4^. Notably, populations at highest risk—heavy smokers, individuals from lower socioeconomic backgrounds, and ethnic minorities—are also those most likely to be under-screened.

Several structural, psychological, and informational barriers contribute to low LCS participation. Structural factors include limited access to screening facilities, transportation difficulties, and financial constraints, disproportionately affecting underserved populations ^4^. Psychological barriers—such as fear of diagnosis, stigma associated with smoking-related diseases, and fatalistic attitudes towards lung cancer—further deter individuals from engaging with screening programmes ^8^. Additionally, informational deficits—including lack of awareness of LCS eligibility criteria and misconceptions about its risks and benefits—reduce uptake ^3^.

Studies in the United States and Europe have highlighted that residents of the most deprived neighbourhoods have higher lung cancer incidence and mortality rates yet are less likely to participate in screening ^9^. This underlines the urgent need for targeted recruitment strategies that address both practical and psychological barriers to LCS participation

***Comparative effectiveness of recruitment strategies***

The effect of recruitment interventions on lung cancer screening participation will be examined using a simplified social ecological classification adapted from Bronfenbrenner’s ecological systems theory ^10^ and McLeroy *et al.*’s framework for health behaviours ^11^. To enhance practical relevance, interventions will be grouped into three categories:

1. **System-level interventions**, such as centralised screening registries and GP-endorsed referrals;
2. **Provider-level interventions**, such as training and decision-support tools; and
3. **Patient-level interventions**, such as personalised invitations, patient navigation, and digital opt-in models.

Research from other cancer screening programmes (e.g., breast and colorectal cancer screening) suggests that combining personalised outreach with patient navigation services is likely to yield the highest participation rates ^12^. However, direct evidence from LCS recruitment remains limited, with most studies focusing on single recruitment interventions rather than comparative effectiveness across diverse populations.

### Epidemiological and economic considerations

The global burden of lung cancer reinforces the need for effective recruitment strategies. An estimated 2.5 million new cases are diagnosed annually, with a disproportionate number of patients presenting at advanced stages ^13^. Early detection through LDCT screening has the potential to significantly improve survival outcomes by shifting diagnoses to earlier, more treatable stages ^11^.

Economically, lung cancer represents a major healthcare expenditure. Cost-effectiveness analyses indicate that well-targeted LCS programmes, particularly those with optimised recruitment strategies, fall within acceptable cost per quality-adjusted life year (QALY) thresholds ^14^. Early detection reduces treatment costs by identifying cancers before they require expensive therapies ^15^. Moreover, targeted recruitment of underserved populations could yield additional economic benefits by reducing the financial burden of late-stage lung cancer treatment.

***Justification for an umbrella review***

The broad range of recruitment strategies available with their respective comprehensive syntheses removes the need for another similar systematic review. Rather, current literature lacks a focus on high-risk subgroups in the context of LCS. Therefore, our plan is to conduct a rigorous review of systematic reviews (i.e., an Umbrella Review) to provide an overview of the existing interventions, and comment on which appear to work best when stratified by population subgroups ^16^.

### Aims and objectives

This umbrella review aims to evaluate the effectiveness of recruitment strategies for lung cancer screening (LCS), particularly among populations with historically low participation rates, via the following specific objectives:

1.   To identify and assess the quality of all systematic reviews examining the efficacy of recruitment strategies for lung cancer screening (LCS).

2.   To classify identified recruitment interventions according to both intervention level (i.e., system-level, provider-level, patient-level), intervention modality (e.g., digital outreach, GP referrals, direct invitations), and healthcare setting (e.g., primary care, hospital-based programmes, regional/national initiatives)

3.   To summarise the evidence on the efficacy of each intervention type, using change in LCS participation rates as the primary outcome.

4.   To stratify the effect of recruitment strategies by population subgroups, such as:

a. Demographic groups (e.g., age, sex, ethnicity)
b. High-risk populations (e.g., heavy smokers, socioeconomically disadvantaged groups)
c. Geographic context (e.g., urban vs rural populations)

5.   To summarise the impact of recruitment strategies on secondary outcomes, including:

a. Screening adherence
b. Knowledge or awareness of LCS
c. Patient experience
d. Barriers and facilitators to participation
e. Cost-effectiveness

6.   To conduct a sensitivity analysis to explore the influence of methodological assumptions regarding:

a. Systematic review quality (e.g., excluding low/critically low-quality reviews)
b. Primary study overlap (e.g., weighting reviews by unique studies or prioritising reviews with stronger synthesis methods)

7.   To produce a narrative synthesis that integrates key insights from the identified reviews, structured by population subgroup, to highlight effective recruitment strategies for each subgroup.

## Methods

### Protocol and registration

This umbrella review follows Joanna Briggs Institute (JBI) guidelines ^17^ and adheres to the Preferred Reporting Items for Overviews of Reviews (PRIOR) checklist ^16^. The proposed methods are pre-registered on the Open Science Framework (osf.io), with further details provided in this a priori publication.

***Eligibility criteria***

Systematic reviews are included if they meet the following criteria:

- **Population:** Patients enrolled in a lung cancer screening (LCS) programme or explicitly eligible for LCS.
- **Exposure/Intervention:** Recruitment strategies designed to increase LCS uptake, such as [direct invitations, media campaigns, digital outreach, or community-based approaches patient navigation, general practitioner (GP) referrals, or digital opt-in models].
- **Comparison:** No recruitment intervention (usual care) or alternative recruitment strategies.
- **Outcome:** Screening uptake (i.e. LCS participation rate).

Eligible studies must be systematic reviews with or without meta-analysis. Scoping reviews, rapid reviews, and systematic narrative reviews will be considered if they meet predefined systematic review criteria, including a clear research question, a reproducible search strategy, eligibility criteria, risk of bias assessment, and systematic data synthesis.

Studies will be excluded if they:

- Do not adhere to standard systematic review criteria.
- Do not focus on recruitment strategies for LCS.
- Assess LCS uptake without evaluating a recruitment intervention.
- Lack a comparison between screening and non-screening groups.

### Information sources and search strategy

A comprehensive literature search will be conducted across MEDLINE (via Ovid), Embase, Scopus, Web of Science, Cochrane Library, CINAHL, and systematic review registries (PROSPERO, Joanna Briggs Institute Database). The search will include studies published from 1 January 2011 up to 31 October 2024 in the English language. Studies dating before 2011 will be excluded as LCS has yet to be established as an evidence-based practice before this time (i.e., before the publication of the National Lung Screening Trial) ^18^.

The search will be structured around three core concepts: (1) lung cancer, (2) screening, and (3) recruitment strategies. Medical Subject Headings (MeSH) terms and free-text terms will be combined using Boolean operators (AND/OR), with filters applied to identify systematic reviews and meta-analyses.

A tailored search strategy will be developed for each database using the SR-Accelerator Polyglot tool to optimise retrieval. Reference lists of included reviews will be hand-searched to identify additional relevant studies ^19^.

### Study selection

Search results will be imported into Rayyan for screening and data management ^20^. Duplicate studies will be automatically removed. Two reviewers will independently screen titles and abstracts, with discrepancies resolved through discussion or third-party arbitration if necessary. Full-text articles will be assessed in the same manner, and the study selection process will be documented using a PRISMA flow diagram ^21^.

***Screening algorithm***

A structured **four-step screening process** will be applied:

1. **Systematic Review Assessment** – Is the article a **systematic review or meta-analysis**? If not, it is excluded.
2. **Lung Cancer Screening Eligibility** – Does the review focus on **LCS-eligible populations**? If not, it is excluded.
3. **Recruitment Strategy Evaluation** – Does the review assess the impact of an LCS **recruitment intervention**on LCS participation rates? If not, it is excluded.
4. **Comparative Studies** – Does the review compare an intervention arm with a control or alternative strategy? If not, it is excluded.
5. **Final Inclusion** – If all the above criteria are met, the study is included.

### Data extraction

A standardised data extraction form has been developed and piloted to ensure consistency and completeness. Two independent reviewers will extract data, with discrepancies resolved through discussion or third-party adjudication. Extracted information will include study characteristics (authors, year of publication, country, type of review, and databases searched), recruitment strategies (description of interventions, mode of delivery, and comparator groups), target populations (general screening populations and high-risk subgroups such as smokers and individuals from lower socioeconomic backgrounds), and reported outcomes. The primary outcome is LCS participation rates. The secondary outcomes are screening adherence, knowledge and awareness of LCS, patient experience, or barriers and facilitators to participation, and cost-effectiveness, where available.

### Data synthesis

The umbrella synthesis will be conducted in three phases:

1. **Summary of Eligible Reviews** – A structured overview of publication details, eligibility criteria, synthesis methodology, and primary study overlap.
2. **Efficacy of LCS Recruitment Interventions** – A focused evaluation of the impact of recruitment interventions on participation rates in lung cancer screening.
3. **Summary of Secondary Outcomes** – A presentation of secondary outcomes in tabulated form, accompanied by a narrative synthesis.

***Phase 1 (Summary of Eligible Reviews)***

Phase 1 will involve a structured summary of the included reviews, conducted in three steps:

(1.1) **Publication Details**: This step will present a tabulated summary of each review’s title, first author, year of publication, country of the first author’s affiliation, and the number of primary studies included.
(1.2) **Review Methodology**: This step will provide a tabulated summary of each review’s eligibility criteria (population characteristics, interventions, and the definition of LCS participation) alongside the synthesis methodology employed (systematic review or meta-analysis). Additionally, a graphical representation of the publication window for the primary studies included in each review will be presented to illustrate the timeframe of the underlying evidence base.
(1.3) **Primary Study Overlap**: This step will present a graphical illustration of primary study overlap. Reviews will be sorted by the end date of their publication window on the y-axis, while the x-axis will display the publication dates of individual primary studies. This visualisation will highlight the temporal distribution of the included evidence and identify patterns of duplication across reviews. If extensive overlap is identified, findings will be weighted to prevent disproportionate influence from frequently cited primary studies.

This structured approach will ensure clear documentation of review characteristics, methodological details, and both the scope and timing of primary studies.

***Phase 2 (Efficacy of LCS Recruitment Interventions)***

Phase 2 will focus on analysing the primary outcome—lung cancer screening participation—through a structured examination of effect estimates. This phase will involve the following steps:

(2.1) **Overall Effect**: The overall effect on LCS participation, as reported by systematic review authors, will be summarised. Where possible, participation rate metrics will be harmonised to ensure consistency across reviews.
(2.2) **Effect Stratified by Review Quality**: Effects will be stratified according to methodological quality assessed using AMSTAR 2. Reviews will be grouped into the four AMSTAR 2 confidence categories (high, moderate, low, critically low) to assess whether high-quality systematic reviews yield consistent or divergent findings compared to lower-quality reviews. Results from reviews assessed as having low or critically low confidence will be highlighted and interpreted with caution.
(2.3) **Effect Stratified by Intervention Level**: The effect of recruitment interventions on lung cancer screening participation will be stratified according to intervention type, using a social ecological classification derived from Bronfenbrenner’s ecological systems theory ^10^ and adapted for health behaviours by McLeroy *et al.* ^12^. Interventions will be categorised as System-Level approaches (e.g., centralised screening registries, GP-endorsed referrals), Provider-Level approaches (e.g., training, decision-support tools), and Patient-Level approaches (e.g., personalised invitations, patient navigation, digital opt-in models).
(2.4) **Effect Stratified by Healthcare Setting**: To examine contextual variation, effects will be stratified by healthcare setting (e.g., national screening programmes, regional initiatives, primary care-led programmes, hospital-based programmes, private services).
(2.5) **Effect Stratified by Subpopulations**: Outcomes will be stratified by relevant subpopulations, including high-risk groups (e.g., heavy smokers, older age groups, socioeconomically disadvantaged populations), demographic characteristics (e.g., sex, ethnicity, urban/rural residence), or other clinically meaningful groups, where data permit.
(2.6) **Narrative Synthesis**: A narrative synthesis will integrate the key insights emerging from this phase of analysis. Where reviews report conflicting evidence, potential reasons for heterogeneity (e.g., variations in intervention methods, target populations, or review methodologies) will be sequentially explored, drawing on the findings of the subgroup analyses where possible.

These analyses will enable the identification of intervention types associated with increased participation in lung cancer screening, while attempting to account for differences in review quality, healthcare settings, and population characteristics, thereby guiding more robust recommendations for LCS implementation.

***Phase 3 (Summary of Secondary Outcomes)***

Phase 3 will consist of cataloguing the secondary outcomes reported across the included reviews. These outcomes will be systematically categorised into the following domains:

- Screening adherence
- Knowledge or awareness of lung cancer screening
- Patient experience
- Barriers and facilitators to participation
- Cost-effectiveness

No additional risk of bias assessment specific to these secondary outcomes will be conducted, as the eligibility criteria of this umbrella review focused exclusively on reviews assessing the primary outcome (participation rates). Since studies evaluating these secondary outcomes independently of participation were likely excluded, a resource-intensive expansion of the risk of bias assessment to an incomplete set of reviews cannot be justified. Instead, Phase 3 provides a structured summary of these secondary outcomes so readers can appreciate the potential ancillary effects, both positive and negative, of interventions aimed primarily at enhancing participation in lung cancer screening.

### Risk of bias assessment

The methodological quality of included systematic reviews will be assessed using AMSTAR 2 ^22^, which evaluates the transparency, reliability, and risk of bias in systematic reviews. Reviews will be rated as high, moderate, low, or critically low quality. For systematic reviews that include meta-analyses, the ROB-ME tool ^19^ will be used to assess the risk of bias due to missing evidence, including selective outcome reporting and small-study effects.

### Sensitivity analysis

Sensitivity analyses will assess the robustness of our findings by testing key methodological assumptions. Additional assumptions may emerge during the review process, and corresponding sensitivity analyses will be added as required. This flexible approach will ensure that the influence of subjective judgment on our conclusions is transparent. However, two sensitivity analyses are anticipated:

• **Quality Assessment**: Results will be compared when excluding only critically low-quality reviews versus excluding both low- and critically low-quality reviews (i.e. when using AMSTAR 2).

• **Primary Study Overlap**: To address primary study duplication, we will explore methods such as weighting reviews by unique studies, excluding reviews with extensive overlap, or prioritising reviews with stronger synthesis methods.

### Certainty of evidence

The GRADE framework will be applied to assess the certainty of evidence for key outcomes ^23^, using the standard classifications of High, Moderate, Low, or Very Low certainty. However, since this is an umbrella review, our GRADE assessments will reflect the quality of the evidence from systematic reviews and meta-analyses rather than direct evaluation of primary studies. The five GRADE domains will be employed:

• **Risk of Bias** will be assessed based on the methodological quality of the included systematic reviews, using AMSTAR 2 and ROB-ME as outlined above. We acknowledge that primary study limitations may not always be adequately reported. Consequently, we will rely on reviewers' assessments of primary study risk of bias where reported and highlight cases where such information is absent or inconsistently described.

• **Inconsistency** will be assessed by evaluating the variability in effect estimates across included reviews. Where meta-analyses are available, heterogeneity metrics such as I² will inform our judgment. For reviews without meta-analysis, inconsistency will be identified based on narrative descriptions of conflicting findings or discordant conclusions across reviews.

• **Indirectness** will be assessed by comparing the characteristics of the included evidence with the population, intervention, and outcome criteria for this umbrella review. Evidence drawn from incomparable screening settings, population subgroups, or outcome definitions may result in a downgrade for indirectness.

• **Imprecision** will be assessed using pooled effect estimates and their confidence intervals where available. Wide intervals that span both meaningful benefit and harm, or intervals that cross clinically important thresholds, will result in downgrading. For reviews without meta-analyses, imprecision will be judged based on the range and variability of reported effect sizes across reviews.

• **Publication Bias** will be considered based on assessments provided in the included reviews. In addition, we will evaluate broader patterns in the data, including unexplained discrepancies between expected and reported findings or trends that suggest unpublished negative results.

## Discussion

LDCT screening has been shown to reduce lung cancer-specific mortality, yet participation rates remain persistently low, particularly among high-risk populations such as smokers, individuals from lower socioeconomic backgrounds, and ethnic minorities. Recruitment strategies vary widely across healthcare systems, but their comparative effectiveness in under-screened populations remains unclear. Existing systematic reviews tend to evaluate participation in cancer screening more broadly, rather than focusing on the unique behavioural and structural challenges associated with LCS uptake ^24^.

This umbrella review will synthesise high-level evidence across systematic reviews to identify the most effective recruitment strategies, highlight knowledge gaps, and inform best practices for improving participation in LCS. By systematically comparing different recruitment approaches, this review will provide critical insights into which strategies work, for whom, and in what contexts.

### Strengths and limitations

Our proposed umbrella review will follow a transparent, systematic approach, adhering to Joanna Briggs Institute (JBI) methodology, the PRIOR reporting framework ensuring methodological rigour. The use of AMSTAR 2 for quality appraisal will strengthen the critical evaluation of included systematic reviews, while citation mapping will help mitigate primary study overlap, reducing the risk of over-representing frequently cited evidence.

A key strength is the comprehensive evaluation of recruitment strategies across diverse healthcare settings, examining system-level (e.g., risk-stratified invitations, automated GP referral mechanisms), provider-level (e.g., clinician training, GP-driven recruitment), and patient-level approaches (e.g., patient navigation, community-based outreach). The integration of the McLeroy 1988 framework ^11^ will also enhance understanding of which strategies are most effective in improving LCS participation, particularly among historically under-screened populations.

However, several limitations must be acknowledged. First, restricting inclusion to English-language publications may limit the generalisability of findings, particularly for LCS programmes in non-English-speaking countries, where recruitment strategies and healthcare systems may differ. Second, excluding grey literature and unpublished studies may result in the omission of emerging recruitment interventions, particularly pilot programmes, implementation studies, and government-led initiatives that may not be formally published. Third, umbrella reviews depend on the quality and scope of existing systematic reviews. If included reviews have methodological weaknesses, inconsistent outcome reporting, or insufficient subgroup analyses, this may limit the strength of the conclusions drawn.

Additionally, heterogeneity in study designs, population characteristics, and healthcare settings may complicate direct comparisons of effectiveness. The application of subgroup analyses and stratification by AMSTAR 2 quality ratings will help address these variations, but residual heterogeneity may remain.

***Implications for future research, policy and clinical practice***

This review will identify and synthesise the literature on the efficacy of LCS recruitment interventions in high-risk subgroups. We aim to highlight underexplored recruitment strategies, such as digital opt-in models, automated risk-stratified invitations, and community-led patient navigation interventions within the 1988 McLeroy framework ^11^.

Our umbrella review of efficacy remains limited in the sense that it does not help us understand “how, in whom and in what specific contexts” the interventions work. A qualitative umbrella synthesis (e.g., utilising a realist approach) could build upon an existing theory. For instance, the I-SAM model could be integrated in this context to propose design of interventions for understudied subpopulations based on a theoretical framework ^15^.

For policymakers, findings will support the development of targeted recruitment frameworks that can be adapted across different healthcare systems. The review will inform evidence-based recommendations for national lung cancer screening programmes, particularly in Europe, where implementation remains fragmented and opportunistic. Insights into cost-effectiveness and system-level barriers will be particularly relevant for countries considering national screening implementation.

For clinicians, particularly those in primary care, findings will offer practical guidance on effective referral pathways and patient engagement strategies. Given that GPs play a key role in recruitment, this review will help identify clinically feasible, scalable approaches to improve uptake. Strategies such as personalised risk communication, automated eligibility identification, and GP-endorsed invitations may emerge as high-impact interventions

## Conclusion

Lung cancer remains the leading cause of cancer mortality, yet participation in LCS remains unacceptably low, undermining its potential to shift diagnoses towards earlier, more treatable stages. Recruitment strategies vary widely, but their effectiveness in different populations and settings remains poorly understood. This umbrella review will synthesise current evidence, assess the quality of existing systematic reviews, and provide evidence-based recommendations for optimising recruitment strategies.

By consolidating findings from multiple systematic reviews, this review aims to provide actionable insights into how recruitment interventions can be tailored to improve uptake, reduce disparities, and enhance the impact of LCS programmes. The findings will have direct implications for policy, clinical practice, and future research, contributing to more effective and equitable lung cancer screening implementation.

## Ethics and consent statement

Ethical approval and consent is not required for systematic reviews.

## Data Availability

No data associated with this protocol. All data supporting this umbrella review will be derived from publicly available systematic reviews and meta-analyses. No new primary data will be collected. The following extended data (i.e., supplementary files) are available via: Open Science Framework, CLIMBER Umbrella Review Protocol, https://doi.org/10.17605/OSF.IO/XVHYT ^25^ This project contains the following underlying data: < CLIMBER_Protocol_PRIORChecklist> PRIOR Checklist for Umbrella Review < CLIMBER_Protocol_PRISMA-P Checklist> PRISMA-P for Systematic Review Protocol < CLIMBER_Protocol_PRISMA Flow Diagram> PRISMA Flow Diagram for Umbrella Review Copyright: © 2025 Cushing B *et al.* This is an open access article distributed under the terms of the Creative Commons Attribution License (CC-By Attribution 4.0 International), which permits unrestricted use, distribution, and reproduction in any medium, provided the original work is properly cited.
