# Supplementary material for: Recruitment Strategies for Lung Cancer Screening: An Umbrella Review Protocol
Source: HRB Open Res. 2025 Jun 25;8:71. [Version 1] doi: 10.12688/hrbopenres.14160.1 (PMC12375178; doi:10.12688/hrbopenres.14160.1)
Supplement: Peer review history

# Reviewer report: Reviewer response for version 1

**DOI:** 10.21956/hrbopenres.15572.r48158

**Published:** 2025-08-23

**Authors:** xiaoshuang feng

**Affiliations:** 1 International Agency for Research on Cancer, Lyon, France, France

This study is going to use an umbrella review study design to examine effective recruitment strategies for improving lung cancer screening participation. The research question is very important given the lung cancer screening programs are being implemented in many European countries. The protocol is clearly described, I have below few minor comments:

1. The European lung cancer screening program introduction didn’t mention UK, where the lung health check is well implemented.
2. It’s not true that Asian countries are lack of organized lung cancer screening, for example, China and South Korea have very large-scale national lung cancer screening programs.
3. Please avoid using “patients” to indicate populations for lung cancer screening, given most of them are not lung cancer patients. Individuals and populations are more appropriate for using.
4. For the study exclusion criteria, I am confused about the point that “Lack a comparison between screening and non-screening groups.” The main research question is update of LCS, not screening effect, why the comparison between screening and non-screening groups are needed here?
5. Given the aim of this work is to be used in the European context, it would be great if consider continents for the stratification analysis.

Is the study design appropriate for the research question?

Yes

Is the rationale for, and objectives of, the study clearly described?

Yes

Are sufficient details of the methods provided to allow replication by others?

Yes

Are the datasets clearly presented in a useable and accessible format?

Yes

Reviewer Expertise:

Lung cancer screening expertise, but not expert in umbrella review study

I confirm that I have read this submission and believe that I have an appropriate level of expertise to confirm that it is of an acceptable scientific standard.

# Reviewer report: Reviewer response for version 1

**DOI:** 10.21956/hrbopenres.15572.r48163

**Published:** 2025-08-20

**Authors:** Fu-Zong Wu

**Affiliations:** 1 National Yang Ming Chiao Tung University, Taipei, Taiwan

Recruitment Strategies for Lung Cancer Screening: An Umbrella Review Protocol

**Introduction** **：**

1. The rationale for an umbrella review is clearly stated; consider specifying inclusion criteria for systematic reviews upfront.
2. Subgroup stratification is valuable; suggest clarifying how overlapping populations across reviews will be handled analytically.
3. Excellent integration of theory; linking Bronfenbrenner’s model to LCS recruitment enhances conceptual clarity and intervention relevance.
4. Aims and objectives are comprehensive; consider prioritizing them or outlining which are exploratory versus confirmatory.
5. Discussion on cost-effectiveness is timely; inclusion of economic outcomes strengthens the potential policy relevance of findings.
6. Please try to discuss more about non-smoker/smokers lung cancer screening uptake rate, heathy awareness and outcome differences.

Reference

 [Ref 1]

 Methods：

1. The protocol is thorough and methodologically sound; consider clarifying how scoping and narrative reviews will be judged.
2. Excellent use of AMSTAR 2 and ROB-ME; suggest pre-defining thresholds for "extensive" primary study overlap.
3. Your GRADE approach is well-articulated; still, clarify how indirectness will be handled for cross-context interventions.
4. The structured screening algorithm improves transparency; a flowchart summarising the steps might help readers follow it.
5. Justify limiting the language to English, given potential bias from non-English systematic reviews on global recruitment.

**Discussion** **：**

1. Clear articulation of strengths; consider elaborating how citation mapping will handle complex overlaps across multi-country reviews.
2. Limiting to English-language and published sources may miss regional strategies—suggest noting this as a source of selection bias.
3. Strong integration of the McLeroy framework; adding an example of a mapped intervention would enhance clarity.
4. Excellent acknowledgement of heterogeneity; recommend stating how residual heterogeneity will be addressed in narrative synthesis.
5. Suggest expanding on how future realist reviews or I-SAM integration could operationalise insights for hard-to-reach subpopulations.

**Conclusion** **：**

1. Strong summary of aims; consider adding a brief remark on limitations to balance the conclusion’s optimism.
2. Suggest clarifying how findings will inform practical implementation, not just future research.
3. Well-positioned implications—adding a sentence on high-risk or underserved populations would reinforce equity relevance.

Is the study design appropriate for the research question?

Yes

Is the rationale for, and objectives of, the study clearly described?

Yes

Are sufficient details of the methods provided to allow replication by others?

Yes

Are the datasets clearly presented in a useable and accessible format?

Yes

Reviewer Expertise:

Toward more effective lung cancer risk stratification to empower screening programs for the Asian nonsmoking population FZ Wu, YC ChangJournal of the American College of Radiology 20 (2), 156-161

I confirm that I have read this submission and believe that I have an appropriate level of expertise to confirm that it is of an acceptable scientific standard.

## References

[1] : Toward More Effective Lung Cancer Risk Stratification to Empower Screening Programs for the Asian Nonsmoking Population. *Journal of the American College of Radiology* .2023;20(2) : 10.1016/j.jacr.2022.10.010 156-161 10.1016/j.jacr.2022.10.010 36646597
